# Outpacing movement — ultrafast volume coverage in neuropediatric magnetic resonance imaging

**DOI:** 10.1007/s00247-020-04771-5

**Published:** 2020-09-19

**Authors:** Daniel Gräfe, Christian Roth, Margit Weisser, Matthias Krause, Jens Frahm, Dirk Voit, Franz Wolfgang Hirsch

**Affiliations:** 1 Department of Pediatric Radiology, University of Leipzig, Liebigstraße 20a, 04103 Leipzig, Germany; 2 Department of Pediatric Surgery, University of Leipzig, Leipzig, Germany; 3 Department of Neurosurgery, University of Leipzig, Leipzig, Germany; 4 Biomedizinische NMR, Max-Planck-Institut für biophysikalische Chemie, Göttingen, Germany

**Keywords:** Brain, Children, Hydrocephalus, Magnetic resonance imaging, Real-time magnetic resonance imaging, Ultrafast magnetic resonance imaging

## Abstract

**Background:**

Conventional MRI sequences are often affected in neuropediatric imaging by unavoidable movements. Therefore, children younger than 6 years usually have to be examined under sedation/anesthesia. A new real-time MRI technique with automatic slice advancement allows for motion-robust T2-weighted volume coverage of the whole brain within a few seconds in adults.

**Objective:**

To evaluate to which extent the new volume coverage method can be used to visualize cerebrospinal fluid and reduce the need for anesthesia in children.

**Materials and methods:**

We assessed 30 children ages 6 years and younger with suspected or proven hydrocephalus, hygroma or macrocephalus using volume coverage sequences with 20 slices per second in three planes. If necessary, a parent was placed in the bore together with the child for calming and gentle immobilization. We compared visualization of cerebrospinal fluid spaces and course of the shunt catheter in volume coverage sequences vs. fast spin-echo sequences.

**Results:**

The clinical issue could be sufficiently assessed in all children with use of volume coverage sequences, whereas conventional fast spin-echo sequences performed moderately to poorly. Visualization of the tip of a shunt failed in 16% of volume coverage scans and 27% of turbo spin-echo scans. A subsequent examination under anesthesia was never necessary. None of the examinations had to be stopped prematurely.

**Conclusion:**

The motion-robust volume coverage sequences with T2-type contrast can be used to avoid sedation of children in the evaluation of cerebrospinal fluid spaces, even in the presence of vigorous motion. For other indications and contrasts, the technique must still be evaluated.

**Electronic supplementary material:**

The online version of this article (10.1007/s00247-020-04771-5) contains supplementary material, which is available to authorized users.

## Introduction

The evaluation of changes in the internal and external cerebrospinal fluid (CSF) spaces comprises a large portion of pediatric MR studies. Repeat examinations are often necessary in children with hydrocephalus, macrocephalus, arachnoid cysts or hygroma. Today, US imaging is the method of choice for children through age 1 year. The usability of US imaging is, however, limited in time by the closure of the large fontanel toward the first birthday, and limited in space by the acoustic window by the parenchyma areas close to the cranial vault and by the decreasing image quality at necessarily lower acoustic frequencies. Thus, to avoid CT with its inherent ionizing radiation, cranial MRI is usually preferred when cross-sectional images are required.

Unfortunately, MRI is generally very sensitive to motion, while smaller children show limited compliance. Therefore, uncooperative children between 2 months and 5–6 years of age can usually only be examined under sedation or anesthesia. However, such procedures are always associated with immediate and delayed risks and further involve high logistic and economic efforts [1]. These aspects need to be balanced against the carcinogenic risk in CT examinations [2]. It would therefore be highly beneficial to abandon anesthesia, especially for recurrent studies and for straightforward clinical issues, if motion-robust and rapid MRI sequences were at hand.

Conventional MRI sequences are constrained by the necessary compromise among spatial resolution, slice thickness, contrast and speed. One solution to bridge these physical limitations is the advances in real-time MRI, which has been able to combine high spatial resolution with extremely high temporal resolution [3]. For example, this technology now allows for dynamic MRI scans of arbitrary movements with up to 50 images per second. A new variant of this real-time MRI method that offers ultrafast coverage of a large volume was recently reported by Voit et al. [4]. However, there have not been any applications in the field of pediatrics, for example, for whole-brain MRI of infants and toddlers. The purpose of the present work was to evaluate its feasibility to study children who were only fixed in the MRI magnet by their parents’ hands for a few seconds, i.e. without the aid of sedation/anesthesia. More specifically, the aim of our study was to assess this innovative volume coverage technique to visualize internal and external CSF spaces and, if present, the position of an intraventricular shunt catheter in small children who would otherwise have required sedation for MRI.

## Materials and methods

We conducted a retrospective analysis of 68 MRI studies from the period between October 2019 and January 2020 using the volume coverage technique with refocused fast low-angle-shot (FLASH) contrast yielding T2/T1-weighted real-time images. All children who were assessed at an age older than 6 years or with clinical questions beyond evaluating CSF spaces were excluded (*n*=38). In the case of repeated examinations in a single child available for analysis, only the first MRI study was included.

All examinations were performed on a 3-tesla (T) MRI scanner (MAGNETOM Prisma^fit^; Siemens Healthineers, Erlangen, Germany). A 64-channel head coil was used. In cases where a mild fixation by the hands of the parent was necessary, the wider 20-channel head coil was applied.

Details of the volume coverage technology have been outlined elsewhere [4]. Essentially, it relies on a heavily undersampled FLASH sequence with radial k-space filling and nonlinear inverse reconstruction with regularization to the preceding frame. To achieve coverage of a large volume, the slice position of each cross-sectional image is advanced by a small percentage of the slice thickness. Although original FLASH sequences are T1-weighted, the present version employed an additional refocusing gradient to ensure T2/T1 characteristics highlighting fluid spaces such as CSF.

**Fig. 1:**
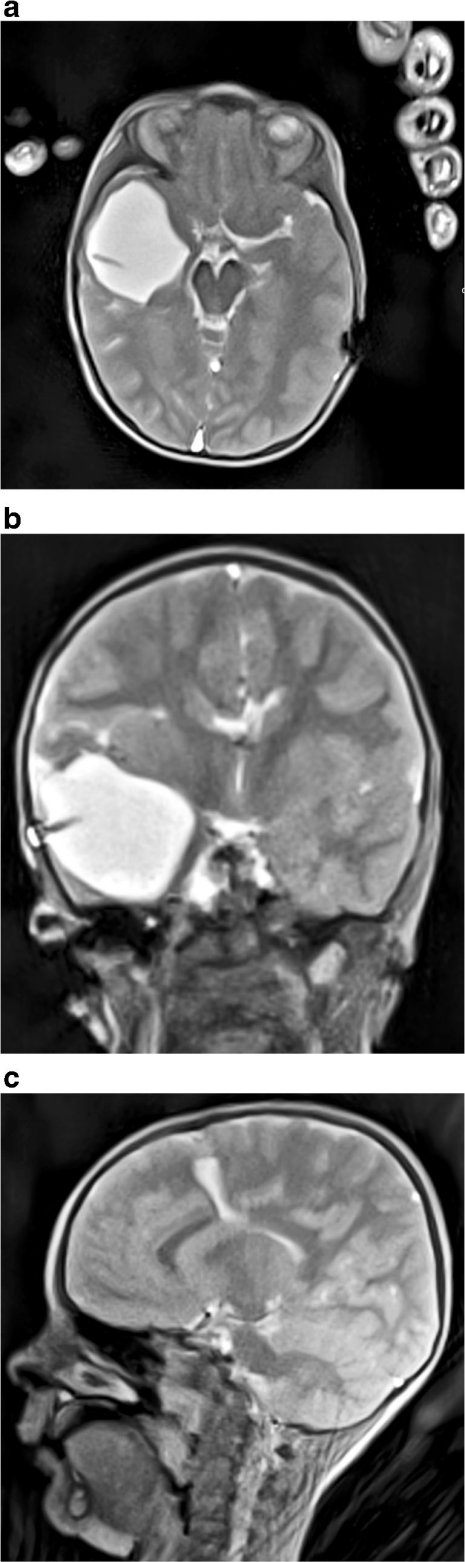
Volume coverage CT in a boy age 5 years 6 months with shunted hydrocephalus, years after relief of a right temporal abscess. **a–c** Volume coverage T2-W sequences are shown in axial (**a**), coronal (**b**) and sagittal (**c**) planes

After a scout sequence in all children, these volume coverage sequences were sequentially applied in three orientations with the following parameters: in-plane resolution 1×1 mm; slice thickness 3 mm; axial, coronal and sagittal planes; flip angle 35°; 13 lines in k-space (spokes) per frame; repetition time [TR]/echo time [TE] 3.85/1.92 ms (Fig. 1). The resulting acquisition time of 50 ms per frame corresponds to a rate of 20 images (sections) per second. By continuously shifting the 3-mm cross-section by a feed rate of 0.45 mm (i.e. 15% of the slice thickness, yielding 85% overlap of successive frames), a total of 200 images per volume coverage scan are generated on average. Accordingly, the total duration of a complete brain examination in one orientation is about 10 s (Supplementary Online Resource 1). The individual 50-ms sections are independent from one another and thus largely resistant to motion.

After acquiring the ultrafast volume coverage sequences, conventional T2-weighted turbo spin-echo sequences (TSE) were recorded for comparison. These TSE sequences had the following parameters: axial plane, in-plane resolution 0.8×0.7 mm without interpolation, section thickness 4 mm, section spacing 4.4 mm, 25 segmented sections, refocusing flip angle 150°, TR/TE 6,390/95 ms, generalized autocalibrating partial parallel acquisition (GRAPPA) 2, turbo factor 26, 5 echo trains per slice, no partial Fourier, acquisition time 38 s. In very anxious or repulsing children, one parent lay down on the examination table in a prone position and covered the child’s earmuffs to achieve gentle immobilization. The duration for the whole examination (scout sequences, shimming, volume coverage, TSE) was about 2:10 min.

The interpretation was based on consensus in simultaneous reporting between two radiologists with 25 years (F.W.H.) and 12 years (D.G.) of experience in pediatric imaging. We evaluated the quality of the volume coverage and TSE sequences using a Likert scale of 1–3 (1=poor, strong artifacts impeding interpretation; 2=moderate, marked artifacts without hindering interpretation; 3=good, at most minimal artifacts). Significance for differences between volume coverage and TSE sequences was determined with the chi-square and Fisher exact tests. Criteria for the success of the ultrafast volume coverage sequences as well as for TSE sequences were sufficient visualization of the internal and external CSF spaces, delineation of a shunt catheter tip, if present, whether the clinical question was successfully answered as well as complete renunciation of subsequent sedation or abortion of the examination. We tracked patients for at least 2 months to determine whether their physicians desired a re-examination or complementary MRI. Our institutional review board approved this retrospective review and we obtained written informed consent from legal guardians.

## Results

**Fig. 2:**
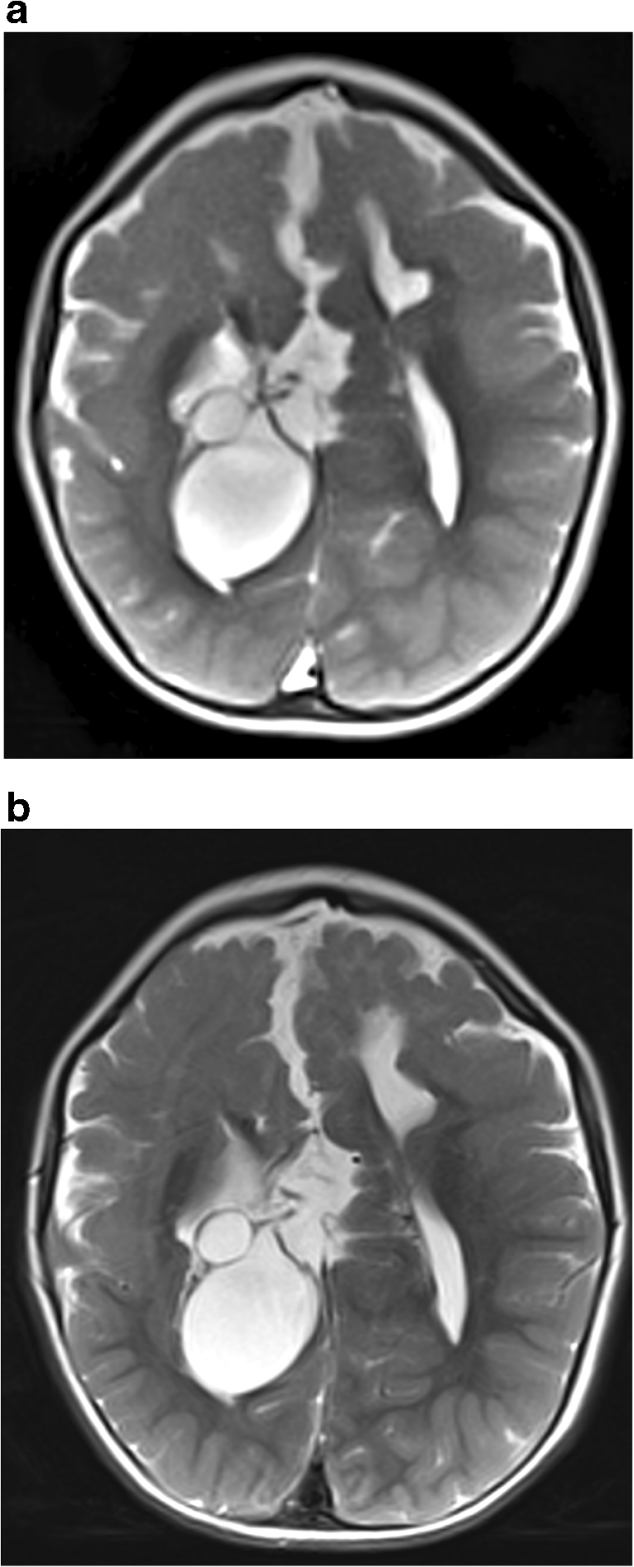
Comparison of axial image quality at rest in a 5-year-old girl with Aicardi syndrome, callosal agenesis and interhemispheric cysts. **a** Volume coverage scan with refocused fast low-angle shot contrast (T2/T1). **b** Conventional T2-weighted fast spin echo

**Fig. 3:**
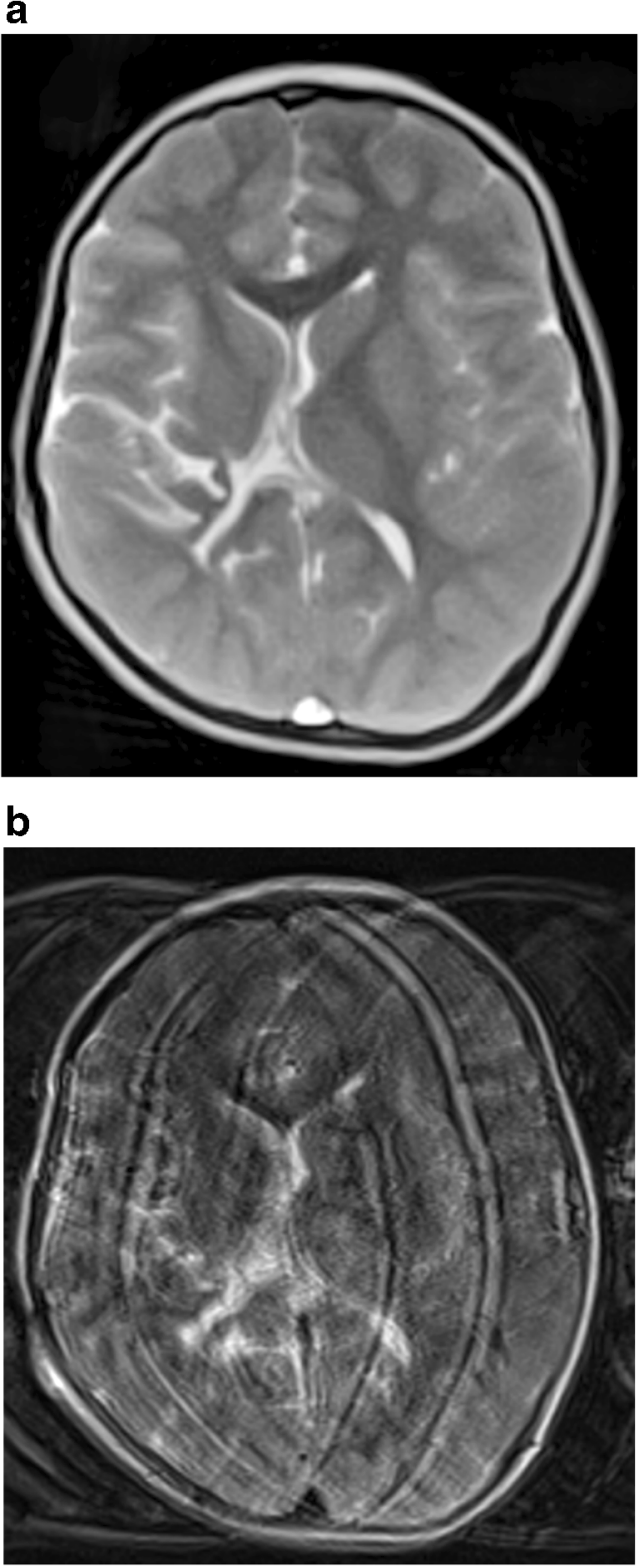
Comparison of axial image quality in the presence of motion in a boy age 4 years 6 months with shunted post-hemorrhagic hydrocephalus. **a** Volume coverage scan with refocused fast low-angle shot contrast (T2/T1). **b** Conventional T2-weighted fast spin echo

The cohort included 30 children between 0.2 months and 75 months (median 32.9 months). Indications for MRI referral were: regular follow-up for hydrocephalus (*n*=13), suspected dysfunction of the ventricular peritoneal shunt (*n*=6), short-term follow-up post-surgery for exclusion of hydrocephalus (*n*=5), exclusion of hydrocephalus (*n*=3), long-term follow-up after hygroma (*n*=2) and macrocephalus (*n*=1). There was no emergency indication in the cohort. No examination had to be aborted prematurely. In every study, the clinical question could be adequately answered without sedation or anesthesia, with volume coverage sequences alone (*n*=30, success rate 100%). In 20/30 children (67%) the infant’s head was softly positioned by a parent. A shunt catheter was present in 19/30 (63%) children; among these, complete visualization of the catheter through volume coverage sequences was achieved in 84% (*n*=16) and through TSE sequences in 27% (*n*=8). Three shunt catheters, especially the tip position, could not be visualized sufficiently with volume coverage (though TSE sequences also failed in these cases). The non-visualization of the shunt catheter in the volume coverage and TSE sequences was irrelevant for the clinical course in our patients. The quality of all volume coverage sequences was almost always good (*n*=29, 97%) (Table 1). Only in one child was the quality in all three planes only moderate (*n*=1, 3%) because of pronounced movement artifacts. The quality of the rapid TSE examinations acquired for reference was good in 47% of cases (*n*=14), moderate in 27% (*n*=8) and poor in 27% (*n*=8). None of the children who were not immobilized by their parents demonstrated a poor TSE exam. Thus, the imaging quality of the TSE reference sequences was clinically sufficient in 73% (*n*=22). The Pearson chi-square test suggested a highly significant difference (*P*<0.001) in the quality ratings between both sequences but had 4/6 cells with an expected frequency of less than 5. A Monte Carlo test with 10,000 repetitions resulted in *P*<0.001. Visualization of the shunt was not statistically different (Fisher exact test: *P*=0.269). Example of the image quality for an uncooperative and for a still child are shown in Figs. 1, 2 and 3.

**Table 1:** Comparison of image quality and visualization of a potential ventriculo-peritoneal shunt between real-time volume coverage sequences versus turbo spin-echo sequences

	Volume coverage	Turbo spin echo	*P*-value
Good quality	29/30 (97%)	14/30 (47%)	<0.001
Moderate quality	1/30 (3%)	8/30 (27%)	
Poor quality	0/30 (0%)	8/30 (27%)	
Visualization of the shunt	16/19 (84%)	12/19 (63%)	0.269

## Discussion

This work describes the first application of a novel ultrafast volume coverage technique yielding motion-robust images at a rate of 20 frames per second. The focus is not primarily on reducing the overall examination time, but rather on achieving the shortest possible acquisition time for each image that indeed resulted in a dramatic reduction or even a complete absence of motion artifacts. Furthermore, the term ultrafast MRI should not be seen in absolute terms, but rather in the context of the current state of MRI technology. For example, in 2000 a single-shot fast spin-echo sequence with an acquisition time of 580 ms per image was considered an “ultrafast” sequence (personal communication, Jens Frahm). As the time required per image has been reduced, it has been proved numerous times in the last 20 years that rapid sequences can be used in children without sedation, such as in half-Fourier acquired single-shot turbo spin echo (HASTE) with 400 ms per image [5], balanced steady-state free precession (bSSFP) sequence with about 250 ms [6] and echoplanar diffusion sequences [7].

In this context, the present volume coverage technique with serial 50-ms sections is by far the fastest T2-weighted neuroimaging approach. Even when the child was lively, there were no common motion artifacts occurring within the image. When the child’s head moved through the plane, only a dark layer appeared in addition to the brief shift of planes (Supplementary Online Resource 2). With a slice overlap of 85% and a slice thickness of 3 mm, this turned out to be clinically irrelevant because the evaluation can exploit a sufficient number of neighboring slices as well as sections from two orthogonal scans. Any in-plane movement, on the other hand, was properly depicted as a movement in serial sharp images (Supplementary Online Resource 3). This is characteristic for the high-speed real-time technology, which apart from other applications has already been employed for cardiac imaging without electrocardiography-gated synchronization [3, 8], for swallowing examinations [9] and for joint movements [10]. In this respect, it is legitimate to also classify the fast T2-weighted refocused brain sequences into the class of real-time imaging.

By using ultrafast real-time volume coverage sequences, we were able to solve the clinical questions completely in all examined children and avoid sedation in 100%. The incomplete visualization of the drainage in three children (16%) was irrelevant for the clinical issue (over-drainage or under-drainage).

Unlike fast spin-echo sequences, the quality of the volume coverage images was fine even with strong movement. In our experience, the approach of employing a parent for gentle immobilization of the child’s head on the MRI table in cases of strong movement has proved to be safe and effective. Bearing in mind all well-known safety precautions, such as preventing contact with the radiofrequency receiver coil and avoiding closed loops and skin-to-skin contact [11], no adverse effects were observed in either the parents or the children.

Notably, the fast spin-echo sequences also produced clinically good or moderate results in 73% of cases. This might be attributed to the fact that the children had already become familiar with the situation because of the preceding volume coverage scans and became gradually more relaxed, although 1 min of scanning time probably does not contribute to calming down. Furthermore, omitting fixation by the parent did not result in poor TSE image quality in any child, probably because of a stronger compliance of the child a priori. However, shunt catheter visualization in TSE sequences was inferior to that in volume coverage sequences (84% vs. 63%), though the difference was not significant.

The assessment of internal and external cerebrospinal fluid spaces is a very common MRI indication in pediatric neuroradiology. Of course, it would be desirable to answer a variety of further questions in uncooperative infants and toddlers. Examples comprise the exclusion of trauma sequelae, the exclusion of intracranial masses, and the postoperative follow-up regarding brain edema and postoperative bleeding. For these applications the volume coverage sequences and their contrast capabilities have to be evaluated separately.

## Conclusion

Motion-robust volume coverage with use of T2-weighted real-time MRI sequences represent a milestone in the pursuit of most gentle neuroimaging of children, especially in comparison to US and CT imaging. Here it has been demonstrated that the evaluation of CSF spaces with this method is feasible even in the youngest children without the need for sedation. Even with an open fontanel, the resulting scans are more informative than cerebral sonography and can be obtained in much less time than a complete US investigation.

## Electronic supplementary material

Supplementary Online Resource 1 Serial volume coverage images with T2/T1 contrast in transverse plane in a 5-year-old girl with Aicardi syndrome, callosal agenesis and interhemispheric cysts (MP4 12448 kb)

Supplementary Online Resource 2 Through-plane motion artifact in a refocused volume coverage sequence in axial plane in a boy age 2.5 years with complex congenital heart disease, thrombosis of superior vena cava and shunted hydrocephalus (MP4 20163 kb)

Supplementary Online Resource 3 In-plane motion artifact in a refocused volume coverage sequence in sagittal plane in a boy age 2.5 years with complex congenital heart disease, thrombosis of superior vena cava and shunted hydrocephalus (MP4 18952 kb)
